# Correlation of transrectal and transabodominal ultrasound measurement of transition zone volume with post-operative enucleated adenoma volume in benign prostatic hypertrophy

**DOI:** 10.11604/pamj.2013.16.149.2532

**Published:** 2013-12-20

**Authors:** Idowu Ajayi, Ademola Aremu, Abimbola Olajide, Tope Bello, Folake Olajide, Victor Adetiloye

**Affiliations:** 1 Radiology Department, Federal Medical Centre, Ido-Ekiti, Ekiti State, Nigeria; 2 Radiology Department, Ladoke Akintola University Of Technology, Ogbomoso, Oyo State, Nigeria; 3 Urology Department, Ladoke Akintola University Of Technology, Ogbomoso, Oyo State, Nigeria; 4 Community Health Department, Obafemi Awolowo, University, Ile-Ife, Osun State, Nigeria; 5 Radiology Department, Obafemi Awolowo University, Ile-Ife, Osun State, Nigeria

**Keywords:** Prostatic hyperplasia, transrectal ultrasonography, enucleated adenoma

## Abstract

**Introduction:**

Benign prostatic hyperplasia is a common disease of ageing men worldwide. Though transrectal ultrasonography (TRUS) is the standard in most parts of the world in evaluation of benign prostatic hyperplasia (BPH), it is rarely done in some less developed countries because of non availability of appropriate probes and or specialists. Transabdominal ultrasonography (TAUS) remains the mainstay in these areas. Some controversies still exist in literature about the accuracy of TAUS evaluation of prostatic volume in patients with BPH. This study aimed at comparing the transition zone volume estimation of the prostate on transrectal and transabdominal ultrasound with post-operative enucleated adenoma volume in Nigeria patients with BPH and to suggest better predictor of prostate volume in evaluation of BPH.

**Methods:**

Forty-six (46) patients with lower urinary tract symptoms due to BPH attending the urologic clinic were evaluated ultrasonographically and eventually managed with open surgery (prostatectomy) after due counselling. The post operative samples were weighted using a sensitive top loading weighing balance and converted to volume. Since the specific gravity of the prostate is equivalent to that of water,the weight is the same as volume.

**Results:**

Patients’ ages ranged between 59 and 90 years with a peak age incidence at seventh decade. Transition Zone (TZ) volume estimation on both transrectal and transabdominal ultrasound showed positive correlation with the post operative enucleated adenoma(r = 0.594, p < 0.001) but the transrectal method was more accurate. There was no significant relationship between the TZ volume and patients’ symptoms(r = 0.491, p = 0.007).

**Conclusion:**

Both TRUS and TAUS are comparable at TZ volume estimation and therefore TAUS can be utilized in regions where intracavitary probes and or the expertise is/are not available.

## Introduction

Benign prostatic hyperplasia (BPH) is a condition intimately related with ageing. Although, it is not life threatening, its clinical manifestation as lower urinary tract symptoms (LUTS) has the potential to reduce patients’ quality of life1. It occurs to a lesser extent in men from middle age onwards and often times characterized by increase in prostatic volume [1]. Presentation in patient with BPH is usually with symptoms of bladder outflow obstruction, but it is known that severity of patients’ symptoms is not a predictor of the size of the prostate gland.

Lower urinary tract symptom is not, however, synonymous with benign prostatic hyperplasia. However, accurate estimation of prostatic volume is an essential step in managing patients with BPH because it bears direct relevance to treatment options offered in each case [2–4]. In clinical practice, pre-operative estimation of prostatic volume is done because prostatic volume bears direct relevance to treatment options available to a particular patient [5, 6].

Studies have been conducted worldwide proving the sensitivity and specificity of TRUS in prostatic evaluation, it delineates the prostatic architecture so clearly that some refer to TRUS as an extension of the urologist finger [7]. Inspite of this, TRUS has remained an untapped tool and underutilized imaging modality for prostate evaluation in our environment [8]. Although, TRUS has assumed an important role in the evaluation of prostate gland pathologies worldwide, some controversies still exist with contrasting reports from various studies [8–10].

We embarked on this study to compare prostate volume estimation by both ultrasound methods with the actual post-operative enucleated adenoma to determine the accuracy of the cheaper and more readily available TAUS, since cost of investigation, availability of equipment and shortage of skilled personnel are the key issues in less developed countries like ours; and also to determine the more accurate method of volume estimation for better patient care in patients with BPH.

## Methods

This is a prospective comparative study carried out at Ladoke Akintola University of Technology Teaching Hospital, Osogbo between June, 2009 and July, 2010.

**Figure 1:**
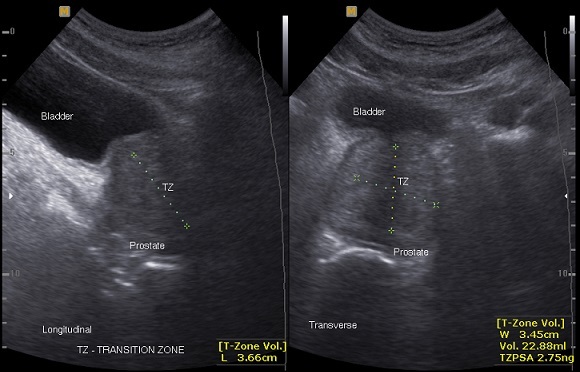
Transabdominal ultrasound of the prostate showing measurement of the transitional zone

After obtaining permission from the ethical and research committee of the institution, consecutive patients presenting in the urology clinic of the teaching hospital with lower urinary tract obstruction (LUTS) were evaluated by same urologist. The patients’ symptoms were assessed and graded using International Prostate Symptoms Score (IPSS). All patients diagnosed with BPH with IPSS = 8 were included in the study. Counselling was done and written consent obtained following which trans-rectal and trans-abdominal ultrasound were done (within 24 hours of open prostatectomy) by the radiologist. The transabdominal scans (Figure 1) were done with the patient's supine after applying ultrasonic gel to the suprapubic regions.

**Figure 2:**
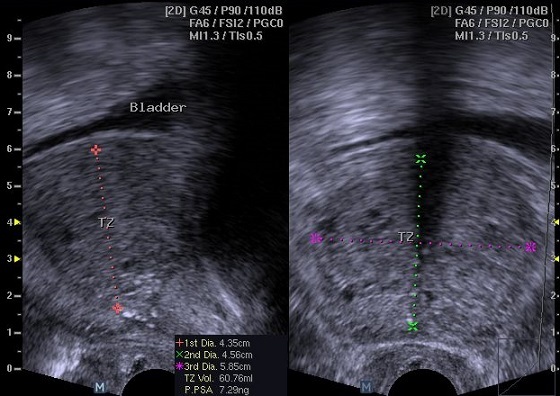
Transrectal ultrasound of the prostate showing measurement of the transitional zone

TRUS was done in Sim's position with an empty rectum and urinary bladder. The latex condom filled with ultrasonic gel was gently introduced into the anus after adequate lubrication with Xylocaine gel. The dimensions of the prostate were measured on both transaxial and longitudinal scans (Figure 2). The longitudinal diameter (Length, L) was measured at the interphase of the prostate with the urinary bladder base. This was done at a slightly off mid-sagittal plane to prevent the urethra from obscuring the cephalad extent of the gland [11]. The transitional zone dimensions were measured from inner boundary of the surgical capsule on saggital and tran-axial scans, the antero-posterior(AP) and transverse(T) were taken on trans axial scans at the widest prostate diameter. The prostate volume was calculated electronically using the ultrasound in built Prolate Ellipsoid Formula (PEF: L x AP x 0.523). The upper urinary tract was assessed by routine abdominal scan for complications.

Excluded from the study were patients that refused to participate, those opting for medical therapy and those with associated anal lesion precluding TRUS like third degree haemorrhoid. All the patients had retropubic prostatectomy done with complete enucleation of the adenoma. Following open retropubic prostatectomy by the surgeon, the specimen was immediately preserved in normal saline to minimize loss of tissue by shrinkage from desiccation and gain of tissue fluid through use of non physiologic fluid. Measurement was done immediately after surgery using a sensitive top loading weighing balance documented to the nearest whole number.

A semi-structured questionnaire was used to collect data on patients’ socio-demographic information, IPSS, ultrasonographic findings (on both TAUS and TRUS), and weight of enucleated adenoma. The data was analyzed using Statistical Package for Social Sciences (SPSS) 16.0 for windows. TZV were correlated in both TAUS and TRUS with enucleated adenoma weight using Pearson's correlation, Regression analysis and paired sample T-test.

## Results

A total of sixty-eight (68) patients were evaluated in the urology clinic that met the criteria for the study. However, only 46 of the patients were operated during the study period.

Their ages ranged between 56 and 90 years with a mean of 69.0 years. Thirty five patients (76%) were in their 7th and 8th decades with about 20% younger than 60 years. The International Prostate Symptoms Score (IPSS) ranged between 14 and 27. Thirty-one patients (67.4%) had IPSS between 9 and 18 while 15 (32.6%) had scores above 19.

Comparison of volume of the transition zone measured on (TAUS), and TRUS with enucleated adenoma is as shown in Table 1. The TZV estimated by both methods correlated well with prostate adenoma weight (p< 0.001).

**Table 1:** Measurement of transition zones on transrectal and transabdominal ultrasound compared with enucleated adenoma

N = 46	Volume/Weight(cm^3^/g)	Mean(cm^3^)	Standard deviation
TRUS TZ	13.58 – 190.05	58.35	41.52
TAUS TZ	10.76 – 186.24	58.79	40.98
Enucleated Adenoma	8.00 – 160.00	62.45	40.91

TRUS TZ : Transrectal ultrasound transition zone ; TAUS TZ : Transabdominal ultrasound transition zone

**Figure 3:**
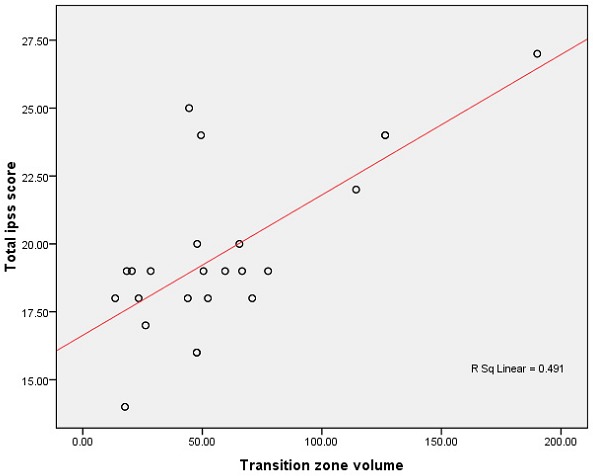
Scattergram comparing the transition zone volume with patient's level of symptoms

The correlation of ultrasound (both TAUS and TRUS) estimated total gland volume and TZV was statistically significant (r = 0.958, p < 0.001) showing a linear relationship between the prostatic size and the transition zone. This implies that the transition zone volume is directly proportional to the total gland volume. However, no relationship is observed between the transition zone volume and patient level of symptoms as shown on the scattergram (Figure 3) which shows several outliers and an analysis of variance (ANOVA) shows poor association with R-squared (R^2^) of 0.491 (p = 0.01). Correlation of the enucleated prostate adenoma weight with the ultrasound estimated volumes revealed moderate significant positive correlation which is higher with TRUS.

During TAUS, 2 (4.3%) patients were noted to have abnormally thickened bladder wall measuring greater than 4mm while 8 (17.4%) patients had simple unilateral renal cysts. Similarly, on TRUS, 4 (8.7%) patients were noted with ejaculatory duct obstruction and another 4 (8.7%) with enlarged median lobe obstructing the internal meatus.

## Discussion

The age of patients in this study ranged between 56 and 90 years with mean age of 69.0 years. Peak age incidence of seventh decade was found which is in agreement with a previous study of prostate Nigeria [2]. This is however contrary to the findings in Europe by de la Rosette and his colleagues who reported peak incidence in the ninth decade [1]. This study also reaffirmed the fact that there is no correlation between TZV and severity of lower urinary tract symptoms due to BPH. Earlier studies have reported similar findings [12, 13]. TAUS is a widely accepted method of imaging the prostate worldwide. This is because it is readily available and more convenient for the patient and the radiologist. TRUS on the other hand is associated with some level of resistance due to its invasiveness and associated discomfort [14]. Patients’ acceptance of TRUS during this study was therefore not without problems especially patients who have had previous TRUS were difficult to recruit for this study. However, all patients eventually consented and cooperated after adequate counselling. Transitional zone was the emphasis in this study because it is the exclusive site of BPH development and it forms the prostatic adenoma enucleated/resected at surgery. The whole prostate volume is only important where radical prostatectomy is intended which is indicated only in patients with early or resectable prostate cancer [8].

The transitional zone volume measured on TRUS and TAUS showed moderate significant correlation (r = 0.594, p < 0.001) with no significant differences in the mean volume although TRUS appeared to be a better predictor of enucleated adenoma when compared to TAUS since it gave a higher correlation value (r = 0.594, p < 0.001). This implied that estimation of pre-operative TZV by both TAUS and TRUS were comparable with insignificant difference. Similar findings were reported by Kim et al and Styles et al who had independent studies done from different centers [9, 10]. This, however differs from latter report by Stravodimos et al which affirm superiority of TRUS over TAUS in accurate prediction of TZV [15]. Baltaci et al compared TZV on TRUS with weight of the enucleated adenoma and found a significant difference in the two parameters [15]. They suggested that the disparity found could be due to irregular adenoma shape or incomplete enucleation at surgery especially in patients undergoing transvesical prostatectomy. They opined that there is improved exposure of the adenoma in retropubic (Millin's) prostatectomy with better inspection of the prostatic fossa, thereby enhancing complete enucleation. We have used retropubic prostatectomy in all the patients in our series to exclude the possibility of incomplete enucleation.

TRUS has an added advantage in the clarity of details seen which is better than TAUS: the surgical capsule is better visualized for easy estimation of the transition zone. Other anatomical details like the ejaculatory ducts, verumontanum and the surrounding prostatic structures especially the seminal vesicles are clearly visualized and better evaluated on TRUS. The heterogeneity of the enlarged transitional zone due to multiple dilated/engorged ductal systems can also be evaluated by TRUS. TRUS is therefore an important tool in evaluation of the male reproductive system. However, concomitant evaluation of the upper urinary tract and other intra abdominal organs as seen with TAUS in this study is a shortcoming for TRUS.

An initial resistance to TRUS was seen in most of the patients,however most of them eventually agreed with good communication and counselling. Also,the low incidence of backpressure effects seen on the upper urinary tracts was misleading and was because most of the patients had been provided with means of relieving the urinary obstruction while awaiting surgery.

## Conclusion

The TZV estimation in patient with BPH is reliable and comparable on both TAUS and TRUS. Although, TRUS is more sensitive (higher correlation with enucleated adenoma) with better clarity, TAUS can be adequately utilized in poor resource regions.
